# Investigation of Cerebral O-(2-[^18^F]Fluoroethyl)-L-Tyrosine Uptake in Rat Epilepsy Models

**DOI:** 10.1007/s11307-020-01503-x

**Published:** 2020-05-14

**Authors:** Carina Stegmayr, Rainer Surges, Chang-Hoon Choi, Nicole Burda, Gabriele Stoffels, Christian Filß, Antje Willuweit, Bernd Neumaier, Alexander Heinzel, N. Jon Shah, Felix M. Mottaghy, Karl-Josef Langen

**Affiliations:** 1 Institute of Neuroscience and Medicine (INM-4; INM-5; INM-11), Forschungszentrum Jülich, 52425 Jülich, Germany; 2 Department of Neurology, RWTH University Aachen, Aachen, Germany; 3 Department of Epileptology, University Hospital Bonn, Bonn, Germany; 4 Department of Nuclear Medicine, RWTH University Hospital Aachen, Aachen, Germany; 5 JARA - BRAIN - Translational Medicine, Aachen, Germany; 6 Centre of Integrated Oncology (CIO), University of Aachen, Bonn, Cologne and Düsseldorf, Germany

**Keywords:** PET, Epilepsy, Rat model, [^18^F]FET

## Abstract

**Purpose:**

A recent study reported on high, longer lasting and finally reversible cerebral uptake of O-(2-[^18^F]fluoroethyl)-L-tyrosine ([^18^F]FET) induced by epileptic activity. Therefore, we examined cerebral [^18^F]FET uptake in two chemically induced rat epilepsy models and in patients with focal epilepsy to further investigate whether this phenomenon represents a major pitfall in brain tumor diagnostics and whether [^18^F]FET may be a potential marker to localize epileptic foci.

**Procedures:**

Five rats underwent kainic acid titration to exhibit 3 to 3.5 h of class IV–V motor seizures (status epilepticus, SE). Rats underwent 4× [^18^F]FET PET and 4× MRI on the following 25 days. Six rats underwent kindling with pentylenetetrazol (PTZ) 3 to 8×/week over 10 weeks, and hence, seizures increased from class I to class IV. [^18^F]FET PET and MRI were performed regularly on days with and without seizures. Four rats served as healthy controls. Additionally, five patients with focal epilepsy underwent [^18^F]FET PET within 12 days after the last documented seizure.

**Results:**

No abnormalities in [^18^F]FET PET or MRI were detected in the kindling model. The SE model showed significantly decreased [^18^F]FET uptake 3 days after SE in all examined brain regions, and especially in the amygdala region, which normalized within 2 weeks. Corresponding signal alterations in T_2_-weighted MRI were noted in the amygdala and hippocampus, which recovered 24 days post-SE. No abnormality of cerebral [^18^F]FET uptake was noted in the epilepsy patients.

**Conclusions:**

There was no evidence for increased cerebral [^18^F]FET uptake after epileptic seizures neither in the rat models nor in patients. The SE model even showed decreased [^18^F]FET uptake throughout the brain. We conclude that epileptic seizures *per se* do not cause a longer lasting increased [^18^F]FET accumulation and are unlikely to be a major cause of pitfall for brain tumor diagnostics.

## Introduction

Positron emission tomography (PET) using amino acid tracers is an established method for brain tumor diagnostics. The response assessment in neuro-oncology (RANO) working group recommends amino acid PET in addition to MRI for the management of patients with brain tumors [1–3] to obtain further information on differential diagnosis, tumor delineation, recurrence diagnosis, and therapy monitoring [4–7].

Epileptic seizure is a frequent onset symptom of brain tumors with an incidence of 20–40 %, while another 20–40 % of patients will experience seizures in the course of the disease. In low-grade tumors, the incidence of seizures can be 80 % and more. While prophylactic anti-epileptic therapy is not recommend, it is mandatory after the first seizures. Seizure control depends on brain tumor treatment and adequate treatment with anti-epileptic drugs. The response rate of patients being seizure-free varies between 30 and 100 % [8–11]. Accordingly, the majority of brain tumor patients investigated with amino acid PET will have experienced seizures prior to the examination.

Recently, Hutterer and colleagues [12] reported about a selected subgroup of ten patients (eight gliomas, one ischemic stroke, one septic encephalopathy) who had widespread and long lasting but finally reversible gyral accumulation of the amino acid analogue O-(2-[^18^F]-fluoroethyl)-L-tyrosine ([^18^F]FET). Initially, the high tracer uptake in those patients was misinterpreted as tumor recurrence, but histological confirmation and follow-up suggested that this accumulation was caused by previous or simultaneous epileptic seizures, accompanied by structural MRI changes. Those ten patients all had either a status epilepticus (SE) or several seizures per day, classified as focal aware or unaware seizures and focal to bilateral tonic-clonic seizures (TCS). Interestingly, [^18^F]FET uptake was detectable up to several weeks after the seizures before it finally decreased.

The aim of this study was to explore whether [^18^F]FET accumulation is a typical and frequent phenomenon in epileptic foci and thus a major pitfall in brain tumor diagnostics. Furthermore, the question arises whether [^18^F]FET PET could serve as a potential marker for epileptic foci. Therefore, cerebral [^18^F]FET accumulation was investigated in two different chemically induced rat epilepsy models. The first, the kainic acid (KA) model, is used as a severe SE model that is known to develop spontaneous seizures a few weeks after the SE due to neuronal loss, reactive gliosis, and mossy fiber sprouting (for an overview, see Dudek at al. [13]). The second, the pentylenetetrazol (PTZ) model, is a kindling model in which the seizures gradually increase over several weeks from mild absences to severe SE. In contrast to the KA model, few structural rearrangements and no chronically recurring seizures are described for this model (for an overview, see Gilbert at al. [14]). Furthermore, the results of the animal experiments were substantiated by [^18^F]FET PET scans of five patients with focal epilepsy and different seizures types within 12 days after the last video-EEG-documented seizure.

## Methods

### Animals

Sixteen male Sprague-Dawley rats (Envigo, Netherlands) were included in this study. All animals were handled in accordance with the Animal Research Committee of the Research Center Jülich GmbH, the German Animal Welfare Act, and the European Community Council directives regarding the protection of animals used for experimental and scientific purposes (2010/63/EU) and with permission by the local authorities (LANUV, North Rhine Westphalia, file number 84-02.04.2017.A281). The rats had a weight of 190–210 g at the beginning of the experiments and were kept under standard housing conditions with free access to food and water, unless they reached a body weight of 350. Food was then restricted to keep that weight.

Six rats underwent titration with kainic acid (KA group), six rats underwent kindling with pentylenetetrazol (PTZ group), and four rats served as controls. After the experiments, the KA rats and two controls were sacrificed for immunohistochemistry.

### Patients

The PET data of five patients with different forms of epilepsy and video-EEG-documented seizures prior to PET investigations (Table 1), who underwent [^18^F]FET PET between December 2017 and March 2019 for the exclusion of brain tumors, were retrospectively evaluated for [^18^F]FET accumulation due to seizures. All subjects had given prior written informed consent for their participation in the [^18^F]FET PET study and evaluation of their data for scientific purposes. The local ethics committee waived the need of approval for evaluation of retrospectively collected patient data. There was no conflict with the Declaration of Helsinki.

**Table 1.** Patient characteristics

Patient	1	2	3	4	5
Sex	F	M	F	M	M
Age	51	60	59	59	32
Diagnosis	TLE of unknown origin with focal aware and unaware seizures	TLE due to a left-temporal ganglioglioma	Bilateral TLE of unknown origin	Bilateral TLE with nocturnal focal unaware and focal to bilateral tonic-clonic seizures	Structural epilepsy with focal to bilateral tonic-clonic seizures due to a left parieto-occipital FCD
Last seizure prior to PET	48 h	36 h	4 days	3 h	12 days
Seizure	Focal unaware seizure	Focal aware seizure	Focal unaware seizure	Focal unaware seizure	Focal unaware seizure
Anti-epileptic drugs	Yes	Yes	Yes	Yes	Yes
Treatment-refractory	Yes	No	Yes	No	Yes
Epilepsy < surgery	No	3× recurrent ganglioglioma	No	No	No
Epilepsy due to limbic encephalitis	No	No	No	Yes	No
Lesion in MRI	No	Yes	No	Yes	Yes

*TLE* temporal lobe epilepsy, *FCD* focal cortical dysplasia

### Induction of Seizures and Timing of Imaging

The experimental setup of the KA group is shown in Table 2. The KA titration was performed in six rats as described elsewhere [15] by repeated i.p. injections of 5 mg/kg kainic acid to induce ≥ 3 consecutive hours of convulsive seizures. Seizures were then stopped by i.p. injection of 20 mg/kg diazepam + 50 mg/kg ketamine [16], and 2 ml/100 g warm lactated Ringer’s solution were injected s.c. to help the rats recover. One rat died, and five rats fully recovered during the next days. In that period, rats were handfed with 1:1 amino acid solution (Aminoplasmal E, B.Braun, Germany) and 30 % glucose solution until they ate normally and gained weight. Four of the five rats had developed spontaneous seizures by 24 days after KA titration. During this period, [^18^F]FET PET and MRI scans were performed regularly.

**Table 2.** Experimental setup of the KA group. Racine class subscriptions refer to the seizure class which the rats exhibited during the previous injections

	SUN	MoN	Tue	Wed	Thu	FrI
W1	KA titration			PET_Racine V_	MR_Racine V_	
W2			MR_interictal_	PET_interictal_		
W3				PET_interictal_		MR_interictal_
W4			Spontaneous seizures in 4/5 rats	PET_interictal/postictal_	Sacrifice	

The experimental setup of the PTZ group is shown in Table 3. After a baseline MRI and [^18^F]FET PET, the PTZ kindling was performed three times per week for 8 weeks by i.p. injection of subthreshold doses of PTZ (20–30 mg/kg BW) to increase seizures from class I in the first week to class V in the last week. During and after the kindling period, [^18^F]FET PET and MRI were performed regularly on seizure-free days. After a pause of several weeks, the still sensitized rats underwent additional 8 days of PTZ injections (one and two injections per day alternatingly), and two [^18^F]FET PET scans were performed on seizure days and seizure-free days, respectively.

**Table 3.** Experimental setup of the PTZ group. Racine class subscriptions refer to the seizure class which the rats exhibited during the previous injections

	Mo	Tue	Wed	Thu	Fr
W0			MR_baseline_	PET_baseline_	
W1	K1		K2	PET_Racine I_	K3
W2	K4		K5	PET_Racine II_	K6
W3	K7		K8		K9
W4	K10	PET_Racine III_	K11	MR_Racine III_	K12
W5	K13		K14	PET_Racine III-IV_	K15
W6	K16		K17	PET_Racine III-IV_	K18
W7	K19		K20	PET_Racine IV_	K21
W8	K22		K23	PET_Racine V_	K24
W9	PET_Racine V_	MR_Racine V_			
W10					
W11			MR_interictal_	PET_interictal_	
W20					PTZ 1
W21	PTZ 2 + 3	PTZ 4	PTZ 5 + 6	PTZ 7	PTZ 8 + 9
W22	PTZ 10	PTZ 11 + 12	PTZ 13 PET_postictal_		
W23	PET_interictal_	MR_interictal_			

*W* week, *K* injection of PTZ for kindling, *PTZ* pentylenetetrazol injection in fully kindled rats

The control rats underwent the same procedures but were injected with saline instead of KA or PTZ.

Severity of seizures was determined based on the Racine’s scale [17] using five stages: (1) hyperactivity, restlessness, vibrissae twitching, mild facial automatisms; (2) strong facial automatisms, head nodding, head clonus, litter pushing; (3) clonic/tonic forelimbs, > 20/10 min body jerks; (4) clonic/tonic generalized seizures with rearing; (5) clonic/tonic generalized seizures with loosing balance.

### PET Imaging with [^18^F]FET

[^18^F]FET was synthesized in-house as described elsewhere with a specific radioactivity of > 200 GBq/μmol [18]. PET imaging in rats was performed under isoflurane anesthesia. A venous catheter was inserted into the rats’ tail vein, and animals were positioned in the field-of-view of the small animal Siemens INVEON scanner (Siemens-CTI) [19]. Body temperature and breathing rate were controlled. After a transmission scan (10 min), dynamic data acquisition was performed in 3D list mode for 47 min starting with injection of 20–30 MBq [^18^F]FET in saline, depending on the rats’ weight, into the tail vein (bolus injection of 0.5 ml in 1 min). Emission data were framed into a dynamic sequence of 2 × 0.5 min, 4 × 1 min, 6 × 3 min, and 6 × 4 min frames. Filtered back-projection (Ramp filter, cut-off = 0.5) was applied to reconstruct 159 slices with an image voxel size of 0.7764 × 0.7764 × 0.796 mm^3^ (matrix size 128 × 128 × 159). Images were corrected for random coincidences, scatter radiation, and attenuation.

All five patients fasted for at least 4 h before PET scanning, according to the German guidelines for brain tumor imaging using radiolabeled amino acid analogues [20]. PET imaging was performed on an ECAT Exact HR+ PET scanner (Siemens Medical Systems, Erlangen, Germany) in 3-dimensional mode (axial field of view, 15.5 cm; image resolution, 6 mm). The dynamic PET studies were acquired in frames of 5 × 1 min, 5 × 3 min, and 4 × 5 min for 40 min after intravenous injection of approximately 3 MBq/kg body weight [^18^F]FET. Iterative reconstruction with 16 subsets and 6 iterations without any filtering was applied; attenuation correction was based on the transmission scan.

### PET Data Analysis

The analysis of the animal data was performed in summed images (18–47 min post-injection) using PMOD (Version 3.902, PMOD Technologies, Ltd.). The PET images of all rats were fitted to PMOD’s Px Rat (W.Schiffer) atlas of rat brain voxel-of-interest (VOIs) regions (Fig. 1). Some VOIs were fused to obtain the mean standardized uptake values (SUV) from the following regions: hippocampus, cerebellum, striatum/thalamus/septum, amygdala/entorhinal cortex, and frontolasso/moto/retrospinal cortex, and the mean of those regions. The MR images of the rats were co-registered to the PET/atlas images to check for correct placement of the rat atlas VOIs.

**Fig. 1.**
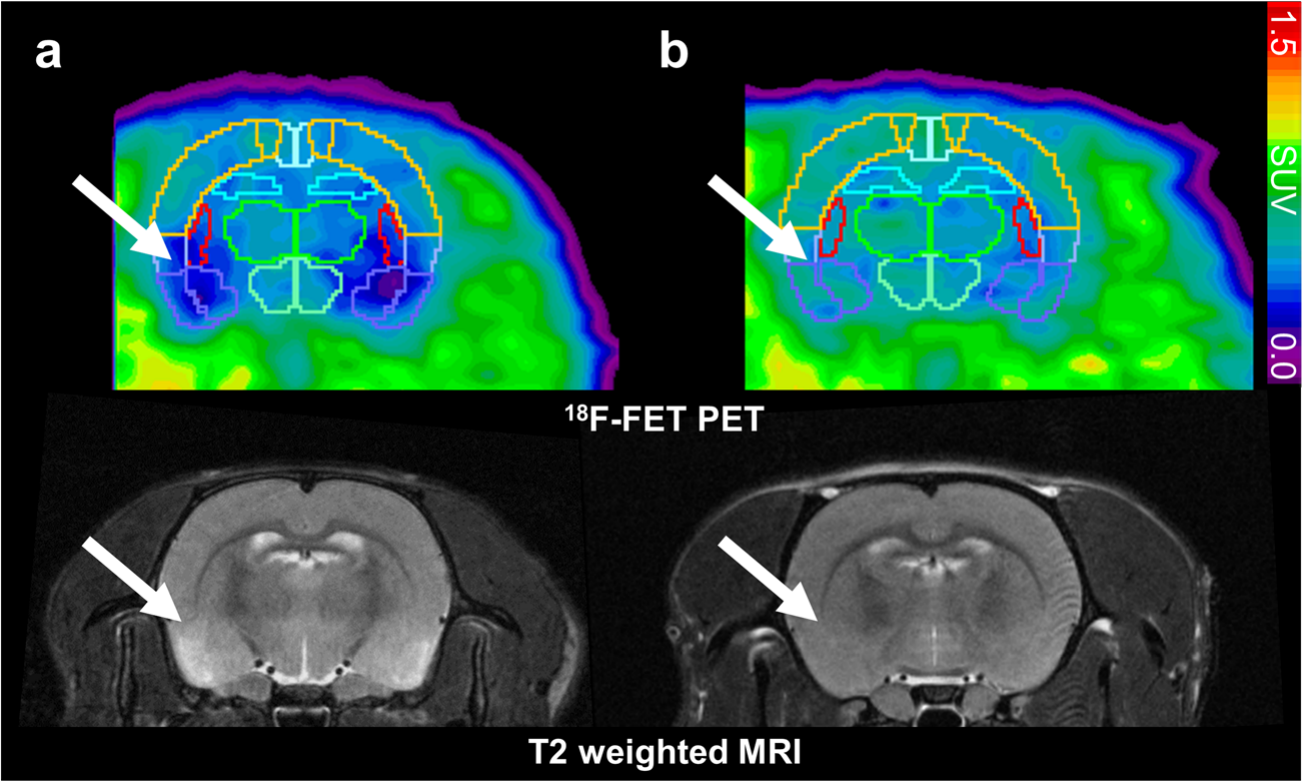
[^18^F]FET PET (upper row) and T_2_-weighted MR scans (lower row) 3d, respectively 4d, after the status epilepticus (**a**) show alterations in the amygdala, piriform, and entorhinal region, compared with a healthy control rat (**b**). Regions of interest: light blue, hippocampus; violet, amygdala and entorhinal cortex; green, thalamus; red, striatum; yellow, fused frontolasso and moto and retrospinal cortex.

The PET scans of the patients were analyzed by visual evaluation of summed images (20–40 min post-injection) for alteration of [^18^F]FET uptake by two experienced nuclear physicians in consensus (G.S., C.F.).

### MR Imaging

The rats were scanned on a small animal 9.4T MRI scanner using an in-house built circularly polarized birdcage rat head coil (diameter of 42 mm) [21]. The MRI scans were started with acquiring scout images and carrying out standard adjustments, such as static magnetic field shimming and radiofrequency power calibration. T_1_ weighted with and without contrast agent and T_2_-weighted images were then acquired using a 3D MP-RAGE and a turbo spin echo sequence, respectively. A venous access connected with a long tubing for contrast agent injection (0.5 ml/kg gadopentetic acid, 0.5 mmol/ml) was inserted into the tail vein. As in PET, rats were anesthetized, and body temperature as well as breathing rate was continuously monitored and maintained.

### Immunohistochemistry

Brains of the KA group, including two controls, were taken out, frozen in isopentane (− 50 °C) and cryo-cut in 20 μm slices coronally. Slices were fixed with paraformaldehyde and subsequently stained for glial fibrillary acid protein (anti-GFAP, DAKO Z0334), microglia (anti-Iba1, abcam ab107159), and neurons (anti-NeuN, Millipore MAB377) using standard protocols for fixed cryoslices. Stained slices of KA-treated rats and healthy controls were evaluated for differences in the visually striking regions determined in PET and MRI.

### Statistics

Descriptive statistics are provided as mean and standard deviation (SD). Two-way repeated measures ANOVAs with all pairwise multiple comparison procedures (Holm-Sidak method) were performed in the KA group as well as in the PTZ group with the factors week of treatment and treatment group (drug or control). Within the KA group, all four vehicle-injected rats could serve as control rats; within the PTZ group, only two of those four rats were suitable as controls. *P* values of 0.05 or less were considered significant. Statistical analysis was performed using the SigmaPlot for Windows, Version 12.5.

## Results

### Kainic Acid Model

All six rats titrated with KA experienced 3 to 3.5 h of consecutive convulsive seizures; one rat died despite the injection with diazepam and ketamine.

In the images of the PET and MR scans 3 days (respectively 4 days) days after the SE, alterations could be detected visually in all five KA rats compared with the healthy controls (Fig. 1). Decreased [^18^F]FET uptake in the amygdala, piriform, and entorhinal region goes along with hyperintensities in T_2_ images in the same regions as well as in the hippocampus. T_1_ images with and without contrast enhancement were observed without pathological finding (data not shown). All visually detectable alterations normalized during the following 21 days and were no longer detectable.

**Fig. 2.**
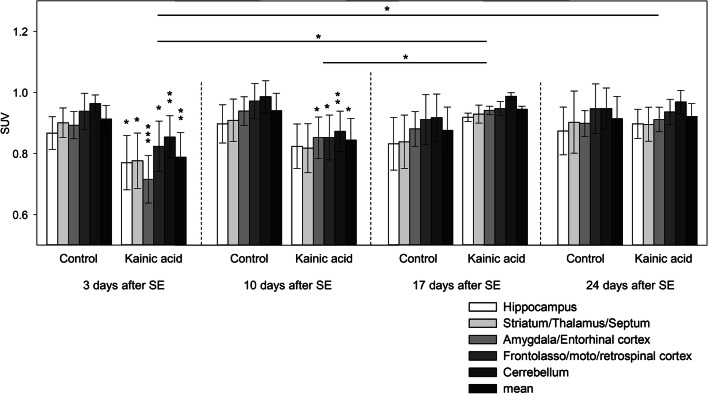
Standardized uptake values (SUV) of [^18^F]FET uptake in the kainic acid model. Three days after severe SE, significantly decreased [^18^F]FET uptake was noted in all examined brain regions compared with control animals which persisted after 10 days (vertical asterisks). After 2 weeks, [^18^F]FET uptake recovered to normal values in all brain regions of epileptic rats (horizontal asterisks). *p ≤ 0.05; **p ≤ 0.01; ***p ≤ 0.001.

Quantitative analysis of PET data (Fig. 2) confirmed decreased [^18^F]FET uptake 3 days after the SE compared with healthy controls. This decrease was most prominent in amygdala, but also significant in all evaluated brain regions. Ten days after the SE, three regions as well as the mean SUV of all regions were still significantly altered compared with the control. In the third and fourth week after SE, the decreased SUV had normalized compared with the controls. Likewise, a significant decrease in all regions was found in the first 2 weeks of the KA group compared with the third and fourth week of the same group (Fig. 2).

**Fig. 3.**
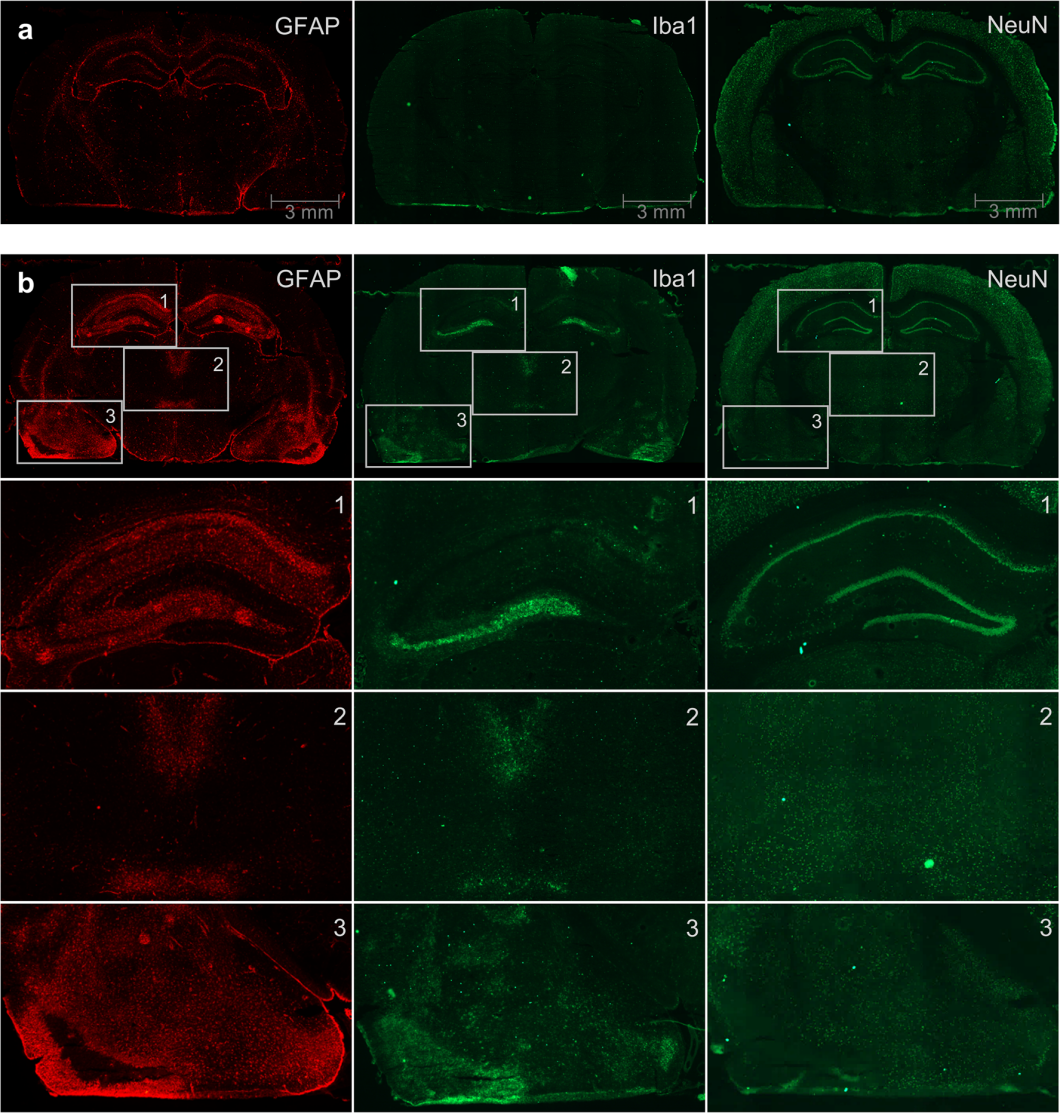
Immunofluorescence staining of a control rat (**a**) and a kainic acid status epilepticus rat (**b**). Consecutive slices were stained for activated astrocytes (GFAP), activated microglia (Iba1), and neurons (NeuN) and visually examined for seizure-related changes. In the hippocampus (1), widespread astrocyte activation is visibly, while microglia are restricted to the lower region (CA3 region) where a loss of neurons was detected. Similar findings—astrocyte and microglial activation accompanied by a loss of neurons—can be detected in thalamic nuclei (intermediodorsal, mediodorsal, reuniens, and ventral thalamic nucleus); (2) as well as in the amygdala and piriform area (3).

The immunohistochemistry of the KA rats showed alterations in the visually striking regions determined from PET and MRI, the hippocampus, and amygdala region, as well as in some thalamic nuclei (Fig. 3). Profound astroglial activation was found in the CA1, CA2, CA3, and dentate gyrus of the hippocampus. Furthermore, the neuronal cell layer of the CA3 and hilus region was lost or severely damaged and showed high microglial activation (Fig. 3(1)). Accordingly, the intermediodorsal, mediodorsal, reuniens, and ventral thalamic nuclei showed astroglial and microglial activation and a decrease of neurons (Fig. 3(2)). In the visually striking amygdala region as defined in PET and MRI, the same widespread and profound astroglial and microglial activation in combination with neuronal loss was detected (Fig. 3(3)).

### PTZ Group

All PTZ-kindled rats developed seizures that increased in severity according to Racine’s scale over 8 weeks. One to two scales per week are listed in Table 3 on the day of imaging which are representative for every PTZ-kindled rat in that week. During the intensive seizure period in week 20–22, most of the seizures were class V, sometimes only class IV seizures occurred. Seizures lasted for < 1 h after PTZ injection, and no spontaneous seizures were observed in between the seizure periods or after the experiment.

**Fig. 4.**
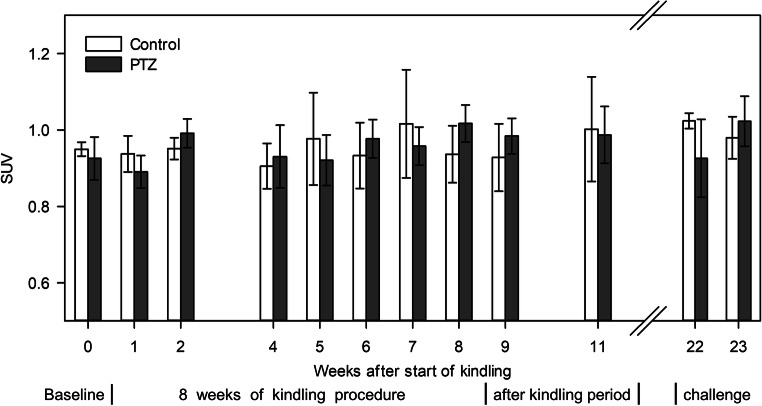
Time line of [^18^F]FET PET uptake in the PTZ model, starting with the baseline scan and ending with the PTZ challenge 23 weeks later. Comparing the SUV of PTZ rats with the control group using a midbrain region containing thalamic, striatal, and septum regions, no alterations in tracer uptake during or after the kindling period or during the PTZ challenge were detected. Likewise, within the PTZ group, no significant changes of the SUV over the time were observed.

In contrast to the KA group, no alterations were observed by visual inspection of PET and MR images (data not shown). Likewise, the quantitative PET evaluation over the whole period of testing did not show significant alterations of the SUV in any tested brain region when comparing PTZ rats with control rats or PTZ rats among themselves over the time. The SUV over the time of a representative midbrain region is shown in Fig. 4.

### Epilepsy Patients

**Fig. 5.**
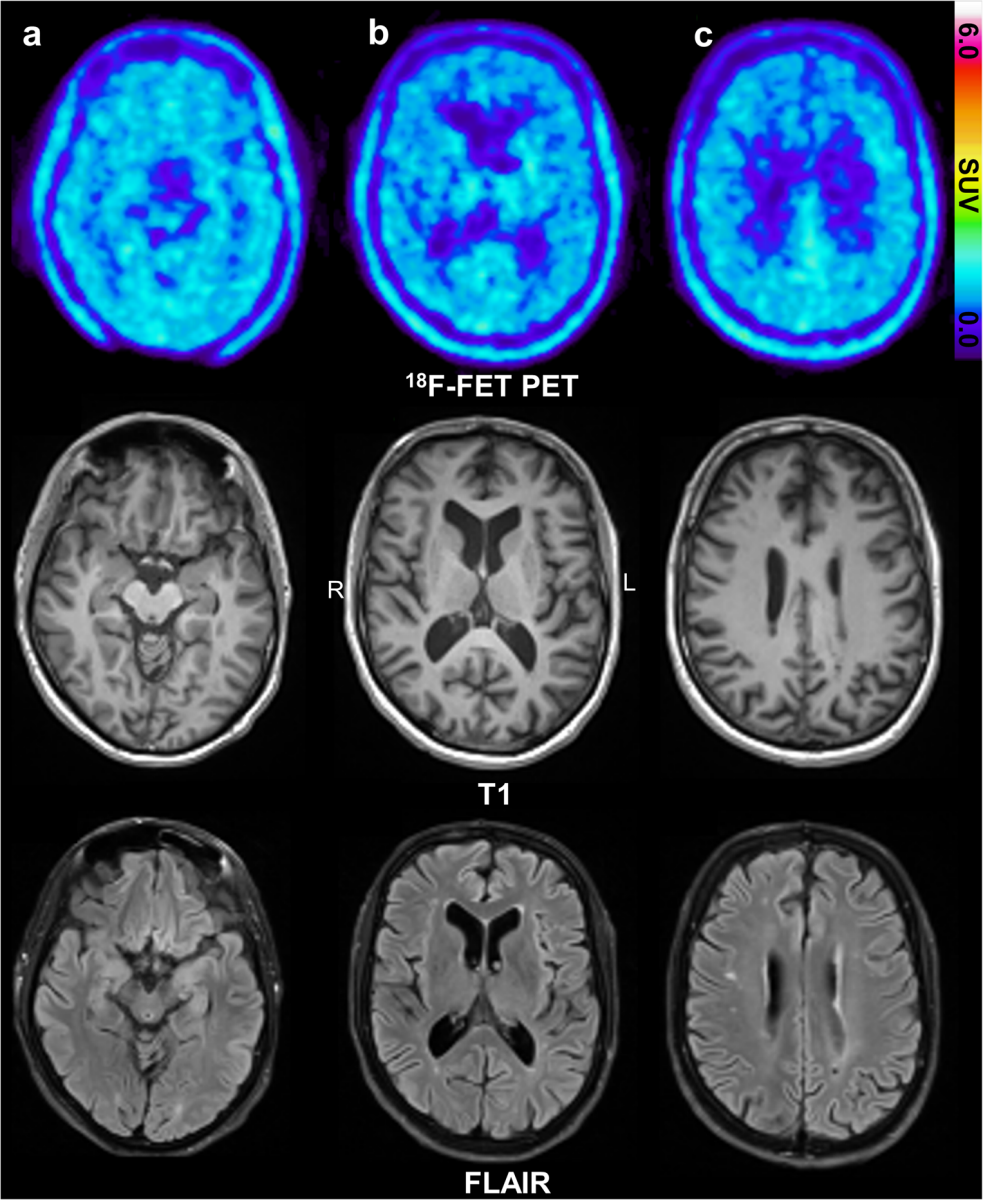
[^18^F]FET PET and MRI of patient no. 4 (Table 1) with bilateral temporal lobe epilepsy with nocturnal focal unaware and focal to bilateral tonic-clonic seizures. No abnormalities in [^18^F]FET uptake are visible. Representative horizontal sections at the level of the temporal lobe (**a**), the basal ganglia (**b**), or just below the centrum semiovale (**c**). Note a small subcortical lesion in the FLAIR sequence in the right hemisphere (**c**).

In four of the five patients, a brain tumor as the cause of the focal epilepsy was excluded. The fifth patient, who was previously operated for ganglioglioma, did not show signs of tumor recurrence. No alteration of cerebral [^18^F]FET uptake, neither increased nor decreased, was detected by visual evaluation in these patients. After the PET results of the animals were available, a second visual evaluation of the patient data was performed with special attention to the hippocampus and amygdala region. Again, no abnormality of cerebral [^18^F]FET uptake was observed. Fig. 5 shows the [^18^F]FET PET and MRI scans of a patient with bilateral temporal lobe epilepsy (TLE).

## Discussion

[^18^F]FET is one of the most widely used amino acid tracers for brain tumor diagnostics. The tracer has a wide diagnostic spectrum, including differential diagnosis, the evaluation of tumor extent, differentiation of tumor progress from treatment-related changes, and therapy monitoring. Therefore, the possibility of unspecific tracer uptake has always been an important field of research [4, 5, 22]. One of those pitfalls which could have considerable consequences for clinical application of [^18^F]FET may be the accumulation in epileptic foci especially in the postictal or interictal phase [12]. This phenomenon has not only been reported for [^18^F]FET, but also for ^11^C-methonine [23–25], the longest established amino acid tracer for brain tumor imaging, which is taken up *via* the same amino acid transporter system LAT1/LAT2 as [^18^F]FET [26–28].

In this study, this issue was systematically investigated, and no evidence for a longer lasting [^18^F]FET accumulation in the postictal of interictal state was found neither in animal models nor in patients suggesting that epileptic seizures *per se* do not appear to be a major cause of pitfall for brain tumor diagnostics.

It has to be discussed to what extent the animal experiments are representative for the clinical scenario in humans. The two rat epilepsy models in our study are chemically induced with systemic injections, but differ in several aspects. KA is an agonist of kainate receptors, one type of inotropic glutamate receptors, and thus leads to excitotoxic lesions, especially in the hippocampus and amygdala [29, 30] (Fig. 3). The spontaneous seizures that occur due to structural rearrangements several weeks after the initial severe SE are considered as a suitable model for chronic epilepsy similar to TLE [13, 31]. Thus, this model was chosen to determine [^18^F]FET accumulation after severe SE in early stage (< 3 days) including disruption of the blood brain barrier (BBB) [32, 33], in the latent period without seizures, and in the beginning of the chronic phase with spontaneous seizures. All phases are accompanied by different levels of structural changes including edema, neuronal loss, and microglia activation. VOIs for quantitative evaluation were located in the hippocampus, amygdala, and entorhinal cortex according to the predominant pathology in the KA model and TLE. VOIs in areas with milder pathology included cortical areas, a midbrain region containing the thalamic nuclei and the septum as well as the cerebellum [34, 35]. We observed no elevated but on the contrary a pronounced decrease of [^18^F]FET uptake 3 days after SE in the amygdala/entorhinal region with concomitant hyperintensities of the T_2_ MRI signal and severe tissue damage, as seen 25 days after the SE by tissue staining (Fig. 3). The hyperintense T_2_ signal indicates vasogenic and cytotoxic edema, a state that peaks around 24 h after KA treatment and subsequently diminishes [36]. In these early hours after the SE, the breakdown of the BBB is most pronounced [32, 37, 38]. In our experiments, we observed no contrast enhancement but more sensitive methods have demonstrated BBB leakage in the KA model as late as 6 weeks after SE [32]. The low [^18^F]FET uptake in our experiments is in opposition to the assumptions made by Hutterer and colleagues [12], who presumed a positive correlation between edema, contrast enhancement, and [^18^F]FET uptake.

In contrast to the KA model, injection of the GABA antagonist PTZ is a kindling model that mimics mild absence to clonic-tonic SE with tissue alterations only in later stages, especially in the hippocampus [39, 40]. Interestingly, and unlike in the KA model, seizures of the PTZ model did not lead to an altered T_2_ signal or [^18^F]FET uptake at any seizure level, although there are reports about altered T_2_ signals in late stages of PTZ-kindled rats [41].

While MR findings indicating an edema normalized during the 4 weeks after the SE regardless of the level of tissue damage, neuronal loss was accompanied by massive microglial and astrocyte activation. According to the neuronal loss especially in the amygdala and hippocampus area (Fig. 3), the recovery of decreased [^18^F]FET uptake to the baseline level in those areas was surprising. A possible explanation might be the microglia and astrocyte activation, which were shown to slightly increase the [^18^F]FET uptake in other pathological states [42]. Decreased uptake due to neuronal loss and increased uptake due to activation processes in the same region cannot be separated in PET due to relatively low spatial resolution and partial volume effects. Hence, uptake appears to be within normal range.

Although the experiments cover the typical pathophysiological changes in cerebral seizures to a very large extent, the animal models have some limitations, *e.g.*, much shorter medical history and generalized seizures due to systemic exposure while patients suffer more frequently from focal seizures, in particular when seizures are tumor-associated. Furthermore, we did not scan any subjects in ictal states; thus, we cannot make a statement concerning short-term [^18^F]FET accumulation in ictal state for patients or rats. Finally, due to few controls (*n* = 2) in the PTZ group, minor changes in the FET accumulation could have been missed. Since the PTZ model is known to show only minor structural changes in contrast to the kainic acid model, it is unlikely that the overall conclusions are affected.

We therefore tried to substantiate the results with patient data and identified five patients in our database who underwent [^18^F]FET PET 48 h to 12 days after seizures for the exclusion of brain tumor-related epilepsy. None of the patients could currently be diagnosed with a brain tumor. Patient diagnosis included TLE with focal aware and unaware as well as focal to bilateral tonic-clonic seizures; two patients were diagnosed with seizures due to structural pathologies. Thus, these diagnoses included some seizure types that may not have been covered by the animal models. No abnormalities of cerebral [^18^F]FET uptake were noted, neither in limbic structures as observed in the animal experiments, nor gyral uptake as reported by Hutterer and colleagues [12].

Thus, the two preclinical models and patient data cover a wide range of seizure classes with and without tissue alterations, and PET scans were performed in interictal as well as postictal states. In no case, increased [^18^F]FET uptake comparable with that in the previous reports was seen and a further elucidation of those findings was not possible. Noticeably, in the existent reports of increased [^18^F]FET and ^11^C-MET uptake, the seizures were in some cases associated with cortical dysplasia [23, 25], which has distinct histological and structural features [43]. We therefore assume that the observed cases in those reports represent a very rare constellation which was not found in the patient population of this study. This is also supported by the fact that in more than 6000 patients examined with [^18^F]FET PET in our institute and in a retrospective study examining long-term epilepsy associated tumors with [^18^F]FET [44], a non-tumor-related, reversible [^18^F]FET accumulation in connection with epileptic seizures has not been noticed.

## Conclusion

Our observation of normal or decreased cerebral [^18^F]FET uptake in two rat models of different grades of seizure and tissue damage and in five patients with different forms of epilepsy indicate that epileptic seizures *per se* do not cause longer lasting increased [^18^F]FET accumulation. Therefore, it is unlikely that [^18^F]FET accumulation due to epileptic seizures represents a major pitfall in the diagnostics of brain tumors. Furthermore, it can be concluded that [^18^F]FET PET is not a suitable method to localize epileptic foci.
